# What constitutes “behavioral health”? Perceptions of substance-related problems and their treatment in primary care

**DOI:** 10.1186/s13722-020-00202-w

**Published:** 2020-07-29

**Authors:** Ida Q. Chen, Helene Chokron Garneau, Timothy Seay-Morrison, Megan R. Mahoney, Heather Filipowicz, Mark P. McGovern

**Affiliations:** 1 Stanford University School of Medicine, Department of Psychiatry and Behavioral Sciences, 1520 Page Mill Road, Palo Alto, CA 94304 USA; 2 Department of Medicine, Stanford University School of Medicine, 1265 Welch Road, Stanford, CA 94305 USA

## Abstract

**Background:**

Integrating behavioral health in primary care is a widespread endeavor. Yet rampant variation exists in models and approaches. One significant question is whether frontline providers perceive that behavioral health includes substance use. The current study examined front line providers’: 1. definition of behavioral health, and 2. levels of comfort treating patients who use alcohol and other drugs. Frontline providers at two primary care clinics were surveyed using a 28-item instrument designed to assess their comfort and knowledge of behavioral health, including substance use. Two questions from the *Integrated Behavioral Health Staff Perceptions Survey* pertaining to confidence in clinics’ ability to care for patients’ behavioral health needs and comfort dealing with patients with behavioral health needs were used for the purposes of this report. Participants also self-reported their clinic role. Responses to these two items were assessed and then compared across roles. Chi square estimates and analysis of variance tests were used to examine relationships between clinic roles and comfort of substance use care delivery.

**Results:**

Physicians, nurses/nurse practitioners, medical assistants, and other staff (*N* = 59) participated. Forty-nine participants included substance use in their definition of behavioral health. Participants reported the least comfort caring for patients who use substances (*M* = 3.5, *SD* = 1.0) compared to those with mental health concerns (*M* = 4.1, *SD* = 0.7), chronic medical conditions (*M* = 4.2, *SD* = 0.7), and general health concerns (*M* = 4.2, *SD* = 0.7) (*p* < 0.001). Physicians (*M* = 3.0, *SD* = 0.7) reported significantly lower levels of comfort than medical assistants (*M* = 4.2, *SD* = 0.9) (*p* < 0.001) caring for patients who use substances.

**Conclusions:**

In a small sample of key stakeholders from two primary care clinics who participated in this survey, most considered substance use part of the broad umbrella of behavioral health. Compared to other conditions, primary care providers reported being less comfortable addressing patients’ substance use. Level of comfort varied by role, where physicians were least comfortable, and medical assistants most comfortable.

## Background

Integrated primary care reflects the ideas of “no wrong door” or “one stop shop whereby patients can received both medical and behavioral health services, including services addressing substance use disorders [1]. Such a model supports, alongside physical health care, the identification, diagnosis, and management of patients with emerging, mild, or moderate behavioral issues including alcohol and other drugs, and refer acute or severe cases to specialists [2, 3]. Clinical research has generated robust evidence for collaborative care models in primary care but have only recently begun to consider substance use disorder treatment and services [1, 4].

Progress is however being made. Interventions such as Medications for Opioid Use Disorder and Screening, Brief Intervention, and Referral to Treatment (SBIRT) have been designed and implemented to various degrees in primary care settings to expand access to substance use services [5, 6]. Whereas only the screening piece of “screening and brief intervention” was routinely implemented in primary care [4], brief intervention is now well executed in at least three large healthcare systems in the United States [4, 7–12]. Further, the Affordable Care Act now funds such care therefore increasing access [13, 14].

Yet, challenges to integrate substance use treatment in primary care settings persist. For instance, the Primary Care–Mental Health Integration (PC-MHI) model of the Veterans Health Administration supports the co-management of primary and mental health care but patients needing substance use services to address anything beyond misuse are referred to specialty services [15, 16]. Further, the Lexicon for Behavioral Health and Primary Care Integration, a standardized manual developed by AHRQ’s Integration Academy to streamline the implementation of integrated behavioral health in primary care settings, mentions substance use throughout but falls short of including concrete implications for policies, services, and workforce requirements necessary for optimal substance use care delivery [1].

Primary care providers are uniquely positioned and vested to partake in integrated behavioral care. Approximately 30% of adult primary care patients have a substance use disorder, yet routine screening and treatment of unhealthy substance use within general practices remains low [17, 18]. This gap in care is staggering given that the epidemiological evidence for comorbidities between substance use disorders, psychiatric disorders, and chronic conditions is well-established and that these comorbidities have been associated with greater mortality, increased health care utilization, and negative patient outcomes [19–23]. A recent study of primary care providers at an integrated VA clinic however found that providers did not view substance use as a focus of their work [24]. Further, primary care providers report unfamiliarity and low levels of preparedness to identify, and assist patients with substance use concerns [25–27].

Behavioral health should include identification and treatment of substance use, but it is unclear if primary care providers and staff believe this to be true and are comfortable with offering identification and treatment of substance use under the umbrella of behavioral health. Data are needed about how much primary care practitioners consider substance use to be within the integrated behavioral health purview. This data can then be used to provide a better understanding of what efforts should be deployed to address gaps for a broader adoption of integrated behavioral health that includes substance use. This study poses the following research questions in an effort to start addressing these gaps: 1. Do primary care providers perceive substance use as an integral part of behavioral health?; 2. Do primary care providers feel comfortable caring for patients who use substances?; and 3. Are there differences in comfort engaging with patients who use substances by clinic role?

## Methods

### Participants and setting

Participants were staff members from two primary care clinics located at an academic medical center in a metropolitan area of Northern California. All staff members with direct patient care responsibilities within these clinics were eligible for participation. Roles with direct patient care responsibilities at these clinic sites include physicians, nurses, nurse practitioners, residents, medical assistants, pharmacists, and administrative personnel. The study sites, a Family Medicine practice and an Internal Medicine practice, are housed in the same building and have a combined panel size of 21,960 patients with an estimated 1015 patient visits weekly.

### Procedure

Data collection took place over a six-week period in April and May 2019. Sixty-five clinic staff members were contacted by email with an invitation to participate in a confidential, online survey about their experience caring for patients. Emails were sent by individuals in leadership positions (i.e. Director of Operations or Medical Directors). This strategy was employed to improve the likelihood that email recipients would complete the survey.

On average, completion of the survey took 10 min. No monetary compensation was provided for participation. All procedures were reviewed and approved by the Stanford University School of Medicine Institutional Review Board. The Institutional Review Board also deemed this study eligible for a waiver of informed consent.

### Measures

The *Integrated Behavioral Health Staff Perceptions Survey* is a 28-item questionnaire developed by the authors of this study, with input and feedback from organizational leadership and care providers. This measure was designed to be a current state assessment tool of providers’ comfort and knowledge of behavioral health, including substance use, as well as time spent on patients with behavioral health needs. Specifically, the instrument covered themes of confidence in clinics’ ability to care for patients’ behavioral health needs, comfort dealing with patients with behavioral health needs, time spent on patients’ behavioral health needs, consistency of care, accessibility to behavioral health care, and provider’s burden caring for patients with behavioral health needs. Participants were also asked to specify what conditions they believed fell under the umbrella of behavioral health. The current report focuses on elements of the survey pertinent to comfort related to patient substance use. Two outcomes were assessed including: perceptions of who is included in provision of behavioral healthcare and comfort caring for patients based on condition.

Clinic role was considered a key independent variable of interest in this study and was measured using a single survey item asking participants to self-report their role. Clinic roles were categorized as: Physicians, Nurses/Nurse Practitioners, Medical Assistants, and “Other.” The “Other” category is comprised of pharmacists, administrative personnel, and those who identified their clinic role as other in the survey. These roles were grouped together in the “Other” category due to their small cell counts. Specific questions from the instrument used for the purposes of this report can be found in Table 1. The *Integrated Behavioral Health Staff Perceptions Survey* is available from the senior author (MM) upon request.

**Table 1:** Selected Items from the Integrated Behavioral Health Staff Perceptions Survey pertinent to this report

Question	Answer
When you think about “Behavioral Health” in the clinic, what kinds of patients do you include? Please select all that apply:	Select all that apply: Patients with mental health problems: such as depression, anxiety, and/or ADHD Patients with substance use problems: such as with alcohol, tobacco, marijuana, opioids–including prescription medications or heroin Patients who are coping with chronic medical conditions: such as diabetes or hypertension, and their adherence to ongoing medical treatment Patient interest in overall health/balance/well-being
Within my role in the clinic, I feel comfortable caring for patients with:	Likert scale, 1 to 5: Strongly Disagree to Strongly Agree Mental health problems Substance use problems Chronic medical conditions and treatment adherence General health concerns
What is your role within the clinic?	Behavioral Health Clinician Medical Social Worker Clinic Manager/Assistant Clinic Manager Nurse Nurse Practitioner Medical Assistant Pharmacist Physician Physician Assistant Other

### Data analysis

Both perceptions of what is included in the provision of behavioral healthcare and comfort caring for patients based on condition were analyzed descriptively and then compared across clinic roles. Chi square tests were used to assess for differences in the inclusion of substance use as a behavioral health issue between study sites, as well as by clinic role. One-way analysis of variance (ANOVA) was used to examine: 1. Differences in level of comfort caring for patients by health condition, and 2. Differences in level of comfort caring for patients who use substances by clinic role. Where appropriate, post hoc mean comparisons were conducted with Tukey’s honestly significant difference (HSD) test. Statistical significance was defined at a *p* value of less than 0.05. Analyses were conducted using Stata, version 13 [28].

## Results

Fifty-nine clinic staff members (91%) participated in the survey. The sample was comprised of 25 internal medicine and family medicine physicians (42%), 17 medical assistants (29%), 4 nurses/nurse practitioners (7%), and 13 other staff members (22%). Twenty-seven participants were from Family Medicine, and 32 participants were from Internal Medicine. There were no significant differences by site for any survey item related to substance use. Participants completed all 28 items on the questionnaire.

### Substance use as behavioral health

**Fig. 1:**
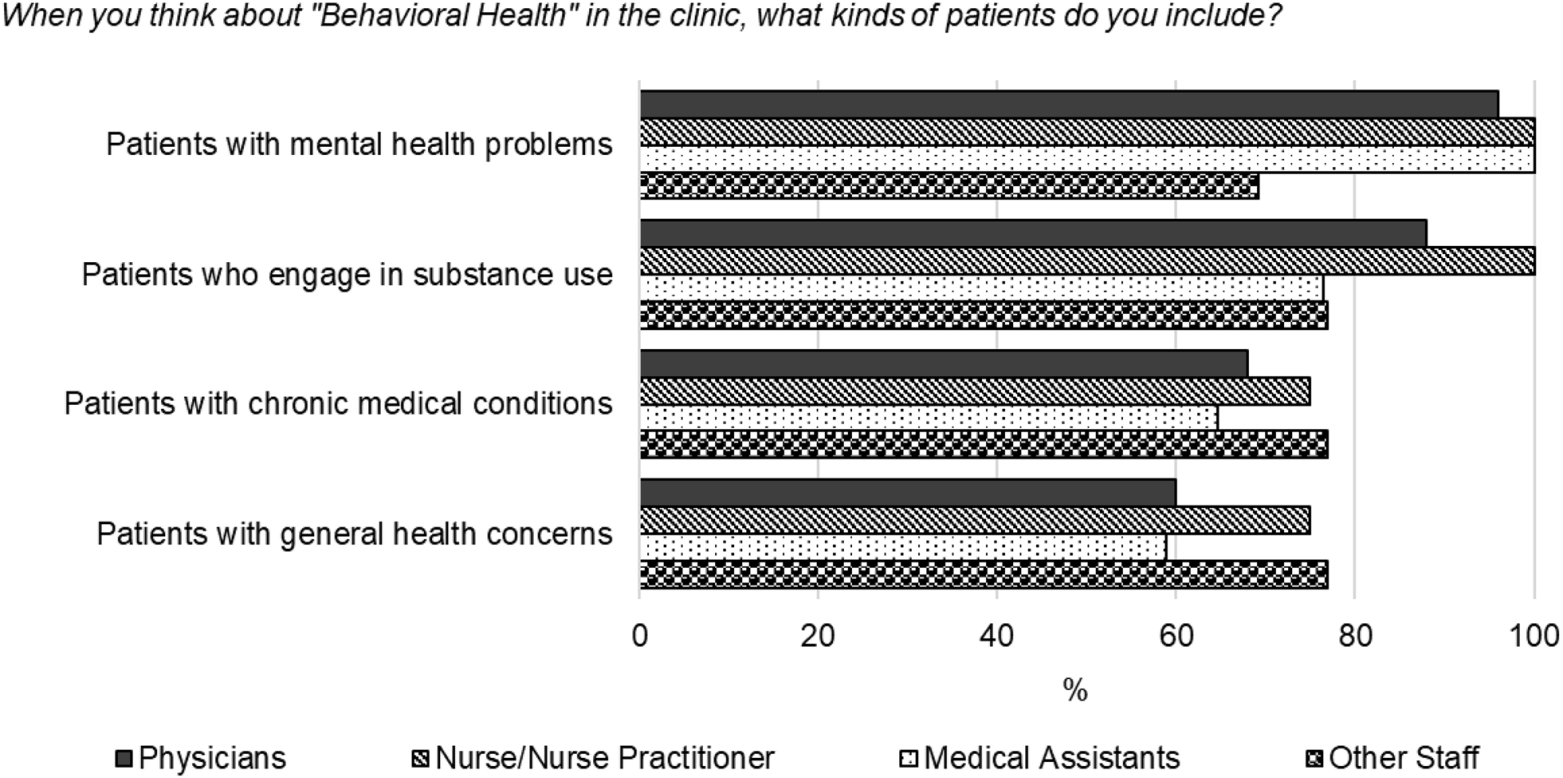
Definition of behavioral health, by clinic role

The majority (*n* = 49; 83%) of participants included substance use in their definition of behavioral health (Fig. 1). No significant differences by clinic role were found in the inclusion of substance use as a behavioral health condition, χ^2^ (3) = 2.12, *p* = 0.548.

### Comfort caring for patients by health conditions

#### Overall

Participants generally reported feeling comfortable caring for patients with four clusters of health conditions: mental health concerns, substance use, chronic medical conditions, and general health concerns.

A statistically significant difference was observed in levels of comfort caring for patients by health condition, *F*(3, 232) = 9.89, *p* < 0.001. A Tukey post hoc test revealed that comfort was lowest for substance use (*M* = 3.5, *SD* = 1.0) compared to mental health concerns (*M* = 4.1, *SD* = 0.7, *p* < 0.001), chronic medical conditions (*M* = 4.2, *SD* = 0.7, *p* < 0.001), and general health concerns (*M* = 4.2, *SD* = 0.7, *p* < 0.001). There were no statistically significant differences between mental health problems and chronic medical conditions (*p* = 0.967), mental health problems and general health concerns (*p* = 0.938), and chronical medical conditions and general health concerns (*p* = 0.999).

#### By role

**Fig. 2:**
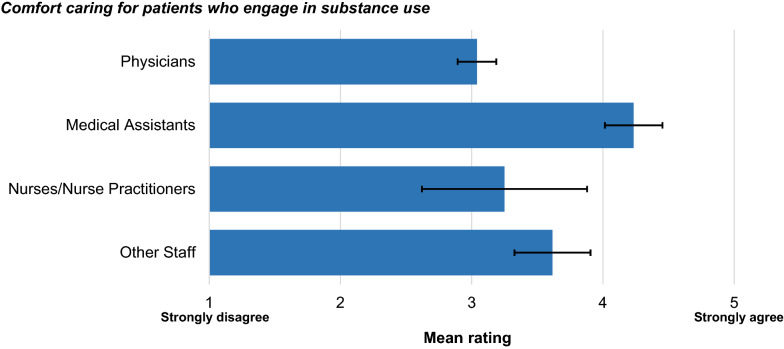
Mean rating for comfort caring for patients with substance use disorder, by clinic role (*N* = 59). Error bars represent standard error

A significant difference by clinic role was found for comfort caring for patients who use substances, *F*(3,55) = 6.22, *p* = 0.001 (Fig. 2). Post hoc comparisons indicate that physicians (*M* = 3.0, *SD* = 0.7) were significantly less comfortable than medical assistants addressing substance use concerns (*M* = 4.2, *SD* = 0.9, *p* < 0.001). In contrast, one-way ANOVA showed no significant differences by clinic role for mental health problems, *F*(3,55) = 2.52, *p* = 0.067; chronic medical conditions, *F*(3,55) = 1.02, *p* = 0.389; and general health concerns, *F*(3,55) = 0.78, *p* = 0.508.

## Discussion

### Summary of findings

With the push for integrated behavioral health services in primary care settings, it is important to understand how primary care practitioners consider patients’ substance use in their conceptualization of behavioral health. Our findings indicate that a majority of care providers consider substance use to be a component of behavioral health. Nevertheless, these same front line providers reported significantly less comfort interacting with patients who use substances compared to patients with mental health problems or chronic medical conditions. With regard to comfort caring for patients with substance use, medical assistants reported greater comfort than did physicians.

## Limitations

Several limitations to this small study are notable. First, the survey was conducted across two clinics within a single health care system, and our findings may not be generalizable to primary care practices in different geographic regions, systems of care, organizations, as well as provider and patient types. Second, the survey was designed for rapid data collection and anonymity so did not gather information on personal and demographic characteristics of staff and providers outside of their role within the clinic. Factors like age, gender, and health care work experience may meaningfully influence perceptions of patients’ behavioral health and substance use. Third, although the response rate was excellent, the sample size is relatively small. Further, there is a possibility that participants’ responses may have been biased given that recruitment emails came from individuals in leadership positions even though confidentiality was ensured.

## Implications

This is the first study to quantitatively examine primary care providers’ conceptualization of integrated behavioral health. Specifically, to assess whether said conceptualization includes substance use. Further, it sought to evaluate comfort caring for patients by health conditions, and by role.

Addressing substance use is critical to the delivery of fully integrated care. Recent studies suggest patients may be more willing to receive behavioral health including substance use treatment services within primary care settings [29, 30]. The high inclusion of substance use in providers’ definitions of behavioral health in our study suggests primary care providers recognize substance use as a behavioral health issue but report discomfort addressing it with patients. This finding is somewhat but not entirely consistent with previously reported findings indicating more negative attitudes toward substance use by non-specialist health professionals [31, 32]. It is however unclear, given that we did not assess for stigma, whether these negative attitudes are about perceived lack of comfort or stigmatization of patients with substance related concerns. Strengthening provider capability and organizational capacity to address these issues, including establishing and reinforcing standardized workflows, can reduce low self-efficacy and improve attitudes about addressing substance use [2]. Differences among provider types is also noteworthy. Physicians reported much lower comfort than medical assistants, who work closely with physicians and play increasingly critical roles in managing care and maintaining patient relationships. This discrepancy may be indicative of differential burden in clinical responsibilities in primary care [33, 34].

## Conclusion

In a small sample of key stakeholders from two primary care clinics who responded to a survey, providers recognized substance use as a behavioral health issue. They however report lower comfort caring for patients who use substances compared to caring for patients with mental health concerns, chronic conditions, or general health concerns. These attitudes may have clinical implications on access to needed care, health outcomes and quality of care. Expanding evidence-based models of integrated behavioral health to include substance use must be a priority in intervention development, evaluating for effectiveness and the potential for implementability in routine practice.

## Data Availability

The dataset generated and analyzed for the current study are available from the corresponding author on reasonable request.

## Abbreviations

AHRQ: Agency for Healthcare Research and Quality
ANOVA: Analysis of variance
PC-MHI: Primary Care-Mental Health Integration
SBIRT: Screening, Brief Intervention, and Referral to Treatment
Tukey’s HSD: Tukey’s Honestly Significant Difference
